# Assessment of the Molecular Responses of an Ancient Angiosperm against Atypical Insect Oviposition: The Case of Hass Avocados and the Tephritid Fly *Anastrepha ludens*

**DOI:** 10.3390/ijms24032060

**Published:** 2023-01-20

**Authors:** Martín Aluja, Mirna Vázquez-Rosas-Landa, Daniel Cerqueda-García, Juan L. Monribot-Villanueva, Alma Altúzar-Molina, Mónica Ramírez-Vázquez, Olinda Velázquez-López, Greta Rosas-Saito, Alexandro G. Alonso-Sánchez, Rafael Ortega-Casas, Adrián José Enríquez-Valencia, José A. Guerrero-Analco, Enrique Ibarra-Laclette

**Affiliations:** 1 Red de Manejo Biorracional de Plagas y Vectores, Instituto de Ecología, A.C.—INECOL, Clúster Científico y Tecnológico BioMimic®, Carretera Antigua a Coatepec 351, El Haya, Xalapa 91073, Veracruz, Mexico; 2 Red de Estudios Moleculares Avanzados, Instituto de Ecología, A.C.—INECOL, Clúster Científico y Tecnológico BioMimic®, Carretera Antigua a Coatepec 351, El Haya, Xalapa 91073, Veracruz, Mexico

**Keywords:** plant–insect interactions, plant defense response to insect eggs, neoplasia, transcriptomics, metabolomics, *Persea americana* cv. Hass, *Anastrepha ludens*

## Abstract

*Anastrepha* spp. (Diptera: Tephritidae) infestations cause significant economic losses in commercial fruit production worldwide. However, some plants quickly counteract the insertion of eggs by females by generating neoplasia and hindering eclosion, as is the case for *Persea americana* Mill., cv. Hass (Hass avocados). We followed a combined transcriptomics/metabolomics approach to identify the molecular mechanisms triggered by Hass avocados to detect and react to the oviposition of the pestiferous *Anastrepha ludens* (Loew). We evaluated two conditions: fruit damaged using a sterile pin (pin) and fruit oviposited by *A. ludens* females (ovi). We evaluated both of the conditions in a time course experiment covering five sampling points: without treatment (day 0), 20 min after the treatment (day 1), and days 3, 6, and 9 after the treatment. We identified 288 differentially expressed genes related to the treatments. Oviposition (and possibly bacteria on the eggs’ surface) induces a plant hypersensitive response (HR), triggering a chitin receptor, producing an oxidative burst, and synthesizing phytoalexins. We also observed a process of cell wall modification and polyphenols biosynthesis, which could lead to polymerization in the neoplastic tissue surrounding the eggs.

## 1. Introduction

Plants and insects have coexisted for millions of years [1]. This long-term interaction has led to an evolutionary arms race, where plants have developed defensive strategies, while insects have evolved to overcome the plants’ defenses [2,3,4,5]. This zig-zag model, which has been described by Jones and Dangl [6], proposes distinct phases, including “pathogen-associated molecular pattern-triggered immunity”; “effector-triggered susceptibility in overcoming pathogen-associated molecular pattern-triggered immunity”; “effector-triggered immunity that further protects the plant against microbial infection”; “countermoves, where the pathogen may evolve to escape recognition by either alteration to the binding specificity of the effector or by the evolution of novel host-defense suppression”. The research on this topic, particularly involving toxic plants defending from herbivore attacks, has been highly productive over the past 50 years (e.g., [7,8,9,10,11,12,13,14,15,16,17,18]). Currently, the molecular tools allow us to unravel the detailed mechanisms that herbivores or plants use during their interactions [19,20,21,22].

In general, the response of plants to herbivory is mostly inducible, and it partially depends on the immediate recognition of the insect [23,24]. This recognition is mediated by specific herbivore-derived molecules known as herbivore-associated molecular patterns (HAMPs) [25]. These molecules can stem from oral secretions, ovipositional fluids, feces, and even herbivore-associated endosymbionts [26,27]. After the HAMPs recognition, the plants trigger a signaling cascade that culminates in the induction of direct and indirect responses [23,28,29]. The direct responses include all of the plant traits involved in enhancing the plant’s resistance against insect herbivores (or other external agents) by damaging the attackers directly. In contrast, the indirect responses involve the mechanisms that do not have a significant impact on the herbivores by themselves, but they can attract natural enemies of the herbivores, significantly decreasing the damage to the plants [20]. Most of these responses are coordinated by the plant’s hormones. For example, after the perception of an herbivory elicitor, the calcium levels are increased in the cytosol, and subsequently, this can modulate the induction of ethylene (ET), jasmonic acid (JA), reactive oxygen species (ROS) and salicylic acid (SA) [22,30]. JA represents a major player in the induction of direct and indirect responses to herbivory [31,32,33,34]. During herbivory, several genes involved in cell wall metabolism and transport are also up regulated, while the genes involved in photosynthesis reduce their expression. This implies that the defense mechanisms are related to the costs and benefits of alternative strategies used by the plants to enhance their fitness [35,36].

In contrast to herbivory, the plant molecular responses to oviposition are poorly understood. The response mechanisms of plants to egg deposition triggers changes in the primary and secondary metabolisms, such as a reduction in the photosynthetic pathway and chemical changes in the plant tissue including an increase in the production of volatile and non-volatile secondary metabolites, which finally induce the formation of neoplasia, egg killing, the call to natural enemies, and/or to a local/systemic preparation for the subsequent larval feeding [30,37,38,39,40,41,42,43,44,45,46,47,48]. It has been suggested that plants can detect the presence of insect eggs through the recognition of egg-associated molecular patterns (EAMPs). Of the few EAMPs that have been identified in eggs, secretions that coat the eggs or mated female extracts, bruchins from the pea weevil *Bruchus pisorum* L., and the cowpea weevil *Callosobruchus maculatus* (F.) (Coleoptera: Chrysomelidae) [41], indole from *Pieris rapae* L. (Lepidoptera: Pieridae) [49], and benzyl cyanide and phosphatidylcholines from *Pieris brassicae* (Lepidoptera: Pieridae) stand out [50,51]. There is also little information on plant-specific receptors of the egg-derived components. In this sense, kinase-like receptors (KLR) play a role during the recognition of egg deposition [44,52]. This is supported by the fact that insect egg extracts, as well as some insect egg-derived lipids, can induce the expression of pathogen-associated-molecular-pattern (PAMP)-genes [52]. In *Arabidopsis thaliana* (L.) Heynh *(Brassicaceae)*, it has been demonstrated that the elicitors from egg extracts of *P. brassicae*, *Spodoptera littoralis* Boisduval (Lepidoptera: Noctuidae), or *Drosophila melanogaster* Meigen (Diptera: Drosophilidae) can induce the expression of some pathogenesis-related (PR) genes, including PR1, a major molecular marker for systemic acquired resistance (SAR), a plant response that results in an increased resistance to virulent pathogens in the distal, unexposed tissues [44,53,54,55]. An egg-killing hypersensitive response (HR)-like necrosis to specialist *Pieris* eggs has also been observed in other members of the Brassicaceae family [48].

In insects, egg survival is considered to be the most relevant factor behind the non-random choice of the oviposition site by females [1,3]. Successful egg development requires an oviposition site that provides appropriate biotic/abiotic conditions, food for the offspring, and a low predation risk [45,46,56,57]. For many insects, the leaves and stems are common oviposition sites [2,4]. In the case of true fruit flies (Diptera: Tephritidae), the eggs can be laid inside the stems, flowers, or fruit (either the pulp or seeds) [58,59]. This pattern has been observed in *Anastrepha*, a fruit fly genus that comprises over 300 described species [60]. Among these, the Mexican fruit fly *Anastrepha ludens* has been widely studied given its wide host range and the fact that it is the most important pest of citrus and mango from Mexico to Costa Rica [61,62].

The ancestral hosts of *A. ludens* are purportedly *Casimiroa edulis* La Llave & Lex. and *C. greggii* (S.Watson) (both Rutaceae), but *A. ludens* can also attack over 15 species of wild and cultivated fruits belonging to the Rutaceae, Anacardiaceae, Rosaceae, Solanaceae, and Lythraceae families [63,64,65,66]. Despite its extreme polyphagy, fitness costs (e.g., delayed larval development and a low pupal weight) have been documented when *A. ludens* attackcertain hosts (e.g., *Malus x domestica* (Rosaceae) [67], *Capsicum pubescens* Ruiz & Pav. (Solanaceae) [56]). In the case of *Psidium guajava* (Myrtaceae), an apparent limit to its polyphagy is reached as it cannot infest this fruit under natural conditions [66], which is likely due to the lack of an association with a key bacteria (*Komagataeibacter*) that is known to degrade/metabolize deleterious polyphenols; tannins are among them [68]. The known mechanisms of resistance of hosts occasionally attacked by *A. ludens* include high levels of polyphenols in apples [67] and laticiferous ducts in mangoes [69]. In the case of *Persea americana* (Lauraceae) cv. Hass (i.e., Hass avocado), Aluja and collaborators [70] demonstrated that this fruit is not attacked in nature by *A. ludens*. However, when *A. ludens* females are forced to lay eggs into commercially ripe fruit, an immediate reaction ensues in the pulp, leading to the eventual formation of a hardened callus (i.e., rigid neoplasia) that completely covers the egg batch, eventually killing them (Figure 1 in Aluja et al., [70]), which is a phenomenon that is similar to the one triggered by the stings of fruit spotting bugs, forming “hard lumps” within the avocado fruit [71,72]. This finding eventually led to the total opening of the US market to Mexican Hass avocados, which had remained closed for over 80 years, and now, it represents one of the most paradigmatic cases of science fostering social and economic growth, as between 2004 and 2017, the exports to the US represented over five billion US dollars and the creation of over 75,000 jobs on both sides of the border [73]. Similar responses have been reported in other plant species. For example, egg deposition by *B*. *pisorum* induces neoplasia formation in *Pisum sativum* L. (Fabaceae) [41]. It has also been suggested that oviposited eggs can trigger the biosynthesis of plant specialized metabolites, with detrimental effects on the eggs. For example, rice produces benzyl benzoate in response to egg deposition by the planthopper *Sogatella furcifera* (Horvath) [38]. However, most of the studies on the plants’ response to oviposition have been conducted on leaves, and the molecular evidence of the plants’ responses to oviposition inside fruits is still scarce.

Considering the insights that this model system could yield for more broadly understanding the response to pests and diseases of this highly sought-after fruit, here, we used microscopic, transcriptomics, and metabolomics approaches to shed light on the mechanisms used by *P. americana* cv. Hass to induce egg killing. To be able to track the molecular changes after oviposition in the fruit of *P. americana* cv. Hass, fruit that had been damaged using a sterile pin (pin) or oviposited by an *A. ludens* female (ovi) were evaluated in a time course experiment considering four different physiological (i.e., fruit ripening) stages: 1 (20 min after damage), 3, 6, and 9 days after the oviposition/pin damage. We also performed an additional time course experiment aimed at describing, with the help of microscopy tools, the physical damage observed in the Hass avocado pulp at 1, 20, and 40 days after the oviposition/pin treatments. Since the Lauraceae represent an ancient plant clade [74], and Hass avocados have had no close association with *A. ludens*, we suggest that its response to the “novel alien” would likely be a general mechanism the plant has developed over millions of years against other diseases and pests [75]. We also predicted that the defensive mechanisms triggered by *A. ludens* oviposition should be mediated by non-specific egg-derived compounds and that it is likely that the response of Hass avocados to *A. ludens* eggs also involves the biosynthesis of specialized metabolites with ovicidal properties.

## 2. Results

### 2.1. Microscopic Analyses

**Figure 1:**
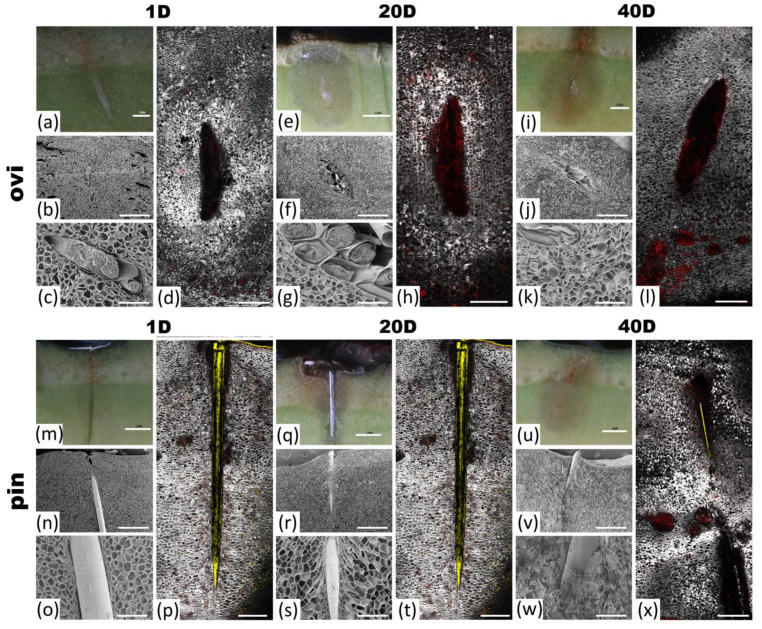
Overview of Hass avocado response to oviposition or pin damage at 1 (20 min after damage), 20, and 40 days after treatments. Visualization of enzymatic browning and necrotic tissue around oviposition (**a**,**e**,**i**) or pin damage (**m**,**q**,**u**) sites. Scanning electron microscopy showed the destruction of cell integrity in **b**,**f**,**j**,**n**,**r**, and **v** (close-up **c**,**g**,**k**,**o**,**s**,**w**, respectively). Confocal images display the cell death (red) by staining of tissues with acridine orange (**d**,**h**,**l**,**p**,**t** and **x**). Bar scales: **a**,**b**,**e**,**i**,**j**,**m**,**n**,**q**,**r**,**u**,**v** = 1 mm; **c**,**f**,**k**,**o**,**s**,**w** = 200 µm; **d**,**h**,**l**,**p**,**t**,**x** = 0.5 mm.

In the time course experiment aimed at describing the physical damage observed in the Hass avocado pulp at 1, 20, and 40 days after the oviposition/pin treatments, we found that immediately after oviposition or the mechanical damage, the tissues began to oxidize, and cell death started around the damaged site (Figure 1). The enzymatic browning of the surrounding tissue started immediately (day 1), but it was clearest at days 20 and 40 after oviposition (Figure 1a,e,i) and the pin damage (Figure 1m,q,u). Cell death (Figure 1d,h,l,p,t,x) and the loss of cell content and integrity (Figure 1b,c,f,g,j,k,n,o,r,s,v,w) were evident at the site of oviposition and the pin damage. This mechanism of response prevented the eggs from hatching and resulted in the death of the eggs possibly because of egg asphyxiation/desiccation or the effect of an ovicidal substance released by the damaged cells and the surrounding ones. Importantly, we found bacteria on the egg surfaces (Figure S1), and this is a phenomenon that we address later because of its possible implications on the signaling pathways that were triggered.

### 2.2. De Novo Transcriptome Sequencing and Data Analysis

As noted in the Materials and Methods section, based on the results of the microscopy study, we repeated the experimental protocol, concentrating our attention on the early stages of the response to the pin and oviposition damage using undamaged fruit as controls. We therefore analyzed/contrasted the transcriptomics changes observed at 1 (20 min after the treatment), 3, 6, and 9 days after the damage. We obtained 482,419,077 paired-end high-quality reads (2 × 150 bp), corresponding to 27 libraries, encompassing a ~324 Giga base (Gb) of raw data. All of the high-quality reads from the 27 RNA-seq datasets were combined and used for the transcriptome assembly using the Trinity assembler (Table S1). A total of 241,009 Unigenes/contigs were produced and cleaned using SeqClean and DeconSeq to obtain a total of 238,568 sequences or unique transcripts. Using AlignWise, 104,244 (43.3%) Unigenes were found to produce peptides of at least 25 amino acids. The redundancy was eliminated using BlastClust, obtaining 101,867 final Unigenes, which were used for further analyses (Table S2).

### 2.3. Differentially Expressed Unigenes (DEGs) in Response to Oviposition and Pin Damage

**Figure 2:**
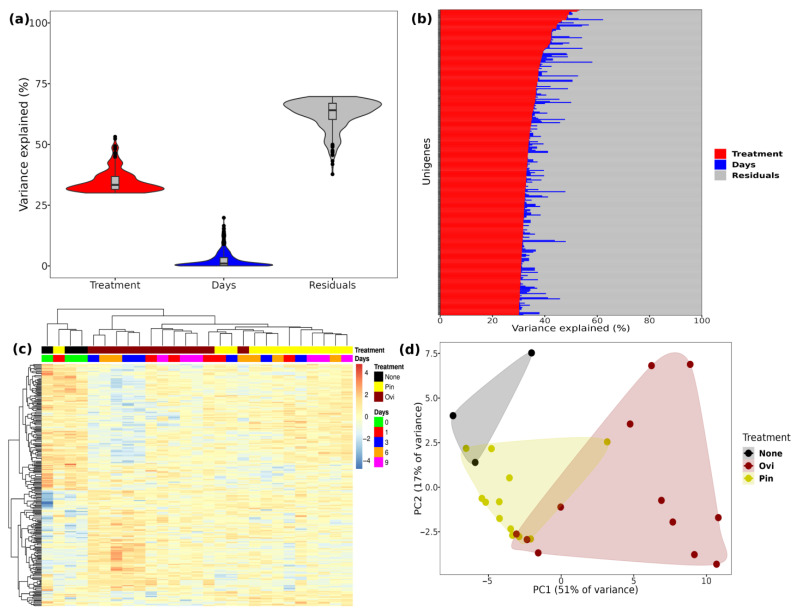
Unigenes related with at least 30% of variance explained by treatment (i.e., undamaged, pin, or ovipositor damaged fruit). (**a**,**b**) Distribution of variance in the 288 Unigenes related to treatment; (**c**) expression profile of Unigenes in treatments during experiment (i.e., 0–9 days, with 0 being related to the undamaged fruit); (**d**) PCA showing the ordination of samples concerning Unigenes related to treatments.

The gene-wise variance partition analysis identified a total of 288 Unigenes associated with between 30 and 53% of the variance explained by the treatments (Figure 2a,b). The sample forms a clear pattern between the treatments and days elapsed after the damage based on these Unigenes, whereas most of the samples clustered together within this group (Figure 2c,d). In the ovi samples, two groups clustered: the first one encompasses days one and nine, and the second one encompasses days three and six. In the linear models of the variance partition, 27 Unigenes were associated with the pin, 150 Unigenes were associated with the ovi, and 111 Unigenes were associated with both of the treatments.

### 2.4. Metabolic Processes Associated with Treatments

**Figure 3:**
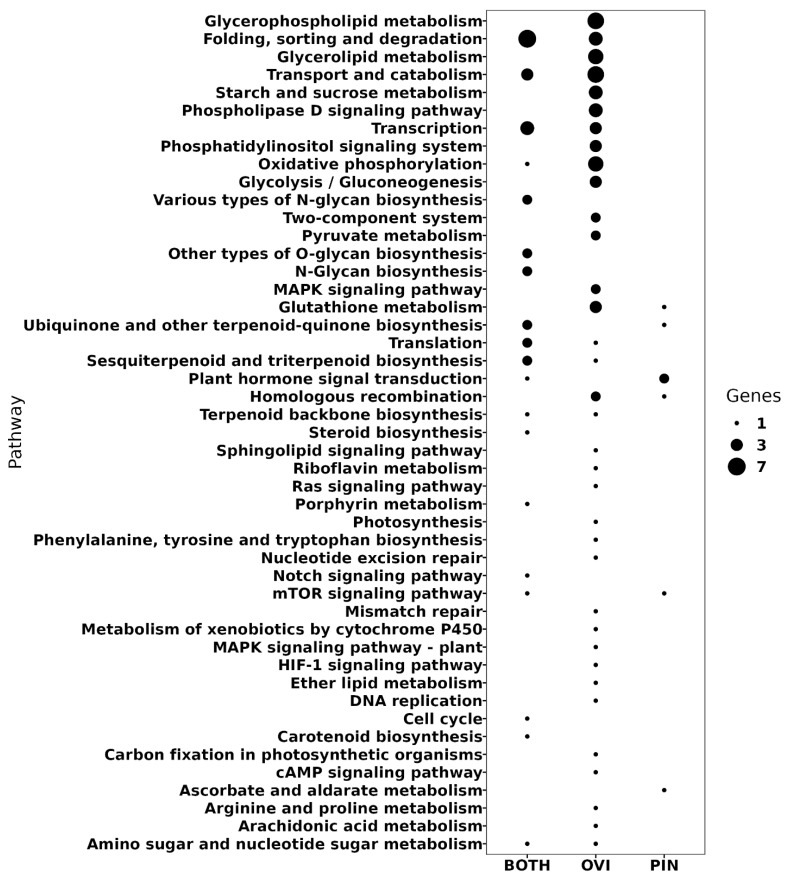
Unigenes mapped in the KEGG pathways. BOTH: Unigenes associated with oviposition and pin treatments; OVI: associated only with oviposition; PIN: associated only with the pin.

We observed that the DEGs exhibited a dynamic behavior within the first days of the experiment, triggering their up or down-regulation mainly between days 1 and 6, but they stabilized after day six, that is, day 9 exhibited a pattern more similar the one observed in the control fruit sampled without being exposed to the treatments (pin or ovipositor insertion). The shared mechanisms of the response to oviposition and pin treatments included: housekeeping metabolism such as cell cycle, transcription and translation, protein folding, sorting, and degradation, and some specialized biosynthetic pathways such as N/O-glycan, ubiquinone, terpenoids, steroids, porphyrin, and carotenoids (Figure 3). Homologs to phospholipase D (UN014960), TTI1 (UN002790), V-type H^+^-transporting ATPase (UN030086), and histone deacetylase (UN029810) were also DEGs identified in both of the treatments. These Unigenes were mapped onto the Mitogen-Activated Proteins Kinase cascades (MAPK) such as the Notch and mTOR signaling pathways. Although the Notch signaling pathway does not exist in yeasts and plants, a functional conservation through the Notchless homolog 1 (NLE) gene, which is conserved in animals, plants, and yeasts, has been suggested. Interestingly, in plants, NLE is involved in numerous developmental processes, including aerial organ size, an increased stomatal index, delayed flowering, and seed development [76,77]. Moreover, NLE also participates in ribosome biogenesis, playing a key role in proper cellular growth and proliferation during plant development [77]. Regarding the Target of Rapamycin (TOR) kinase, its role as a key developmental regulator in both plants and animals has been documented [78]. In all eukaryotes which have a functional TOR kinase, this protein integrates the environmental and nutrient signals to direct growth and development. Despite the lack of information about how TOR is involved in different developmental processes, recent studies have shown that it is involved in plant development from embryogenesis to senescence [77,78]. We noticed that although the avocado Unigenes which codify for NLE and TOR homologs (UN003046 and UN029464, respectively) were not identified as DEGs, their presence in the Unigene set was supported by a high number of transcripts.

Regarding the different responses between the pin and oviposition treatments, we found that the Unigenes identified as DEGs only in the pin treatment correspond (among other functions) to a mechanosensitive ion channel (MSL1, ID: UN016990), which was up regulated upon the pin damage. In addition, the metabolism of ascorbate and aldarate and two genes coding for transport inhibitor response 1 (TIR1, ID: UN004700) from plant auxin signal transduction were also identified. In contrast, a basic endochitinase B (ChiB, ID: UN047162) of the MAPK signaling pathway of the plants was triggered by the forced oviposition. Moreover, the DEGs that only respond to the oviposition were mapped to 26 KEGG pathways. The oviposition-associated pathways with up-regulated DEGs relate to the homologous recombination mechanism and the arachidonic acid metabolism, arginine and proline metabolism, amino sugar and nucleotide sugar metabolism, metabolism of xenobiotics by cytochrome P450, cAMP signaling pathway, ether lipid metabolism, Ras signaling pathway, sphingolipid signaling pathway, oxidative phosphorylation, two-component system, pyruvate metabolism, and starch and sucrose metabolism. In contrast, the oviposition-associated pathways with down-regulated DEGs were riboflavin metabolism, sesquiterpenoid and triterpenoid biosynthesis, carbon fixation, HIF-1 signaling pathway, terpenoid backbone biosynthesis, phosphatidylinositol signaling pathway, glycerolipid metabolism, photosynthesis, phenylalanine, tyrosine, and tryptophan metabolism, mismatch repair, and nucleotide excision repair.

The other pathways associated with oviposition showed both up and down-regulated DEGs such as glutathione metabolism, with one down-regulated (leucyl aminopeptidase, ID: UN026683) and two up-regulated genes (glutathione peroxidase and glutathione S-transferase, ID: UN109125 and UN102508); glycerophospholipid metabolism, with one up-regulated (phospholipase D1/2) and two down-regulated genes (diacylglycerol kinase, ID: UN007801 and UN012748); glycolysis/gluconeogenesis, with two up-regulated genes (pyruvate kinase, ID: UN026537 and UN027952) and one down-regulated (phosphoglycerate kinase, ID: UN071737) gene. Moreover, two auxin response factors (ARF, ID: UN019777 and UN019080) with a B3 DNA-binding domain (DBD) were exclusively down-regulated in the oviposition treatment.

**Figure 4:**
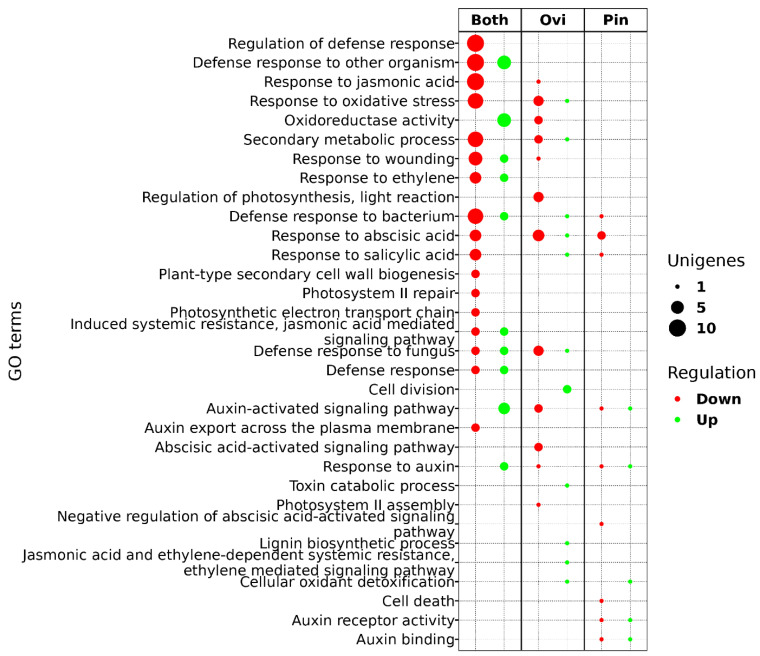
The DEGs classified by the Gene Ontology categories. The dot size shows the number of Unigenes in each category, and the color indicates if the UniGene was up- regulated (green) or down- regulated (red) in oviposition (ovi), pin, or both of the treatments.

We classified the DEGs with their *Arabidopsis* homologs (Table S3) to identify their gene ontology categories related to hormone regulation, secondary metabolism biosynthesis, defense response, and cell death, and expansion mechanism, resulting in 32 GO terms (Figure 4, Tables S4 and S5). Most of the shared DEGs between the treatments were related to regulation of the defense’s response category and responses to hormones such as JA, ET, auxin, abscisic acid (ABA), and SA. The GO terms specific to the oviposition were JA and ET-dependent systemic resistance, ET-mediated signal pathway (UN047162: basic chitinase), toxin catabolic process (UN102508: glutathione S-transferase tau 7), cell division (UN025758 and UN042665: UDP-N-acetylglucosamine (UAA) transporter family), and lignin biosynthetic process (UN049167: cinnamyl alcohol dehydrogenase 9). It suggests that the oviposition by *A. ludens* triggers an immune-response-like reaction once the eggs are detected.

### 2.5. Metabolites Associated with Oviposition and Pin Damage

**Figure 5:**
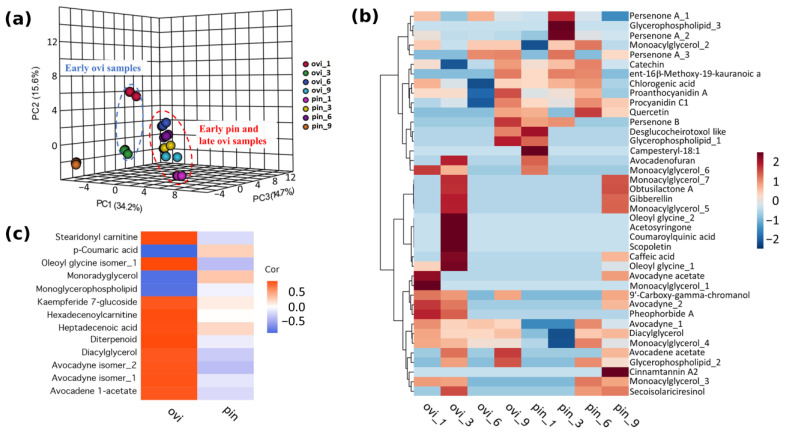
Metabolomics analysis of oviposition- and pin-treated samples. (**a**) PCA analyses of the metabolomic profile of samples at 1, 3, 6, and 9 days after each treatment. (**b**) Heatmap of differentially accumulated compounds between pin and ovi treatments. (**c**) Heatmap of metabolites correlated with the DEGs only in the case of the oviposition treatment.

**Figure 6:**
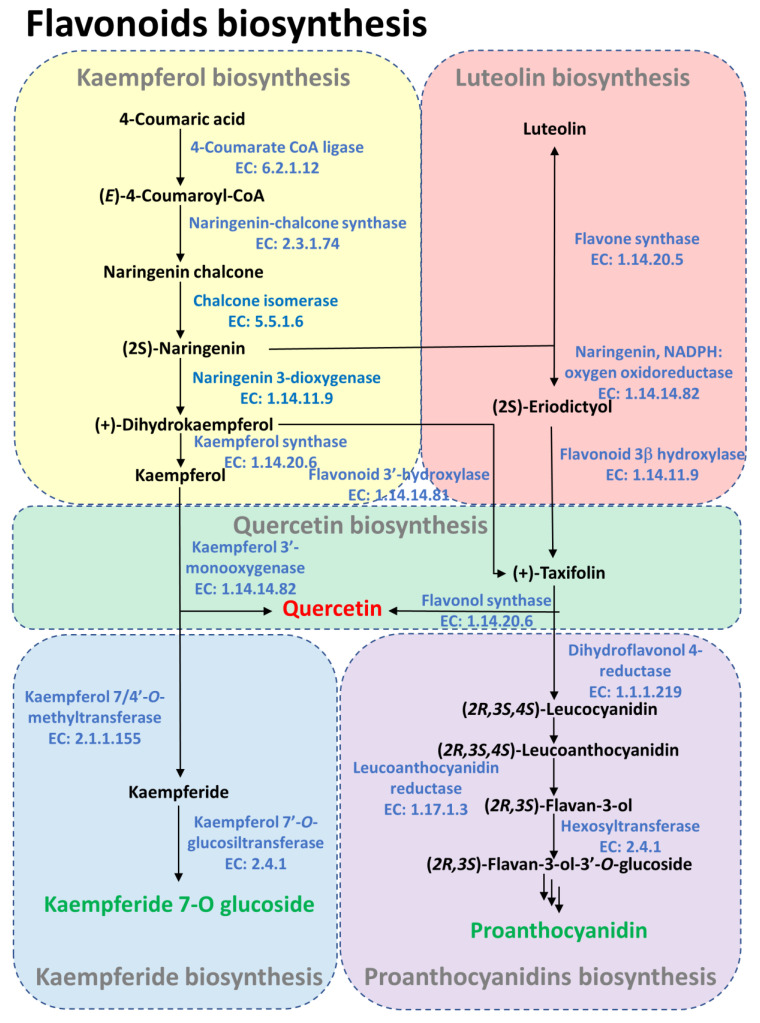
Flavonoid biosynthetic pathway involved in pin and/or ovi treatments. Enzymes and compounds are in blue and black, respectively. Triple arrows indicate a multi-step enzymatic conversion. The avocado Unigenes annotated as homologs/orthologs are shown in Table S8. Bioactive compounds accumulated in pin or ovi treatments are in red and green, respectively.

Our results indicate that the response of the Hass avocado to the damage caused by the insertion of the aculeus of the ovipositing female is likely induced by the recognition of a biological agent. Therefore, to expand our knowledge on the chemical changes induced by oviposition, we performed a metabolomics analysis. The untargeted metabolomics analysis flushed out a clear chemical difference between the oviposited and pinned samples, mainly in the early oviposited samples (ovi_1 and ovi_3) and the late pinned sample (pin_9; Figure 5a). Interestingly, the early pinned and late oviposited samples remain similar in the three-dimensional principal component analysis, due to their similar chemical composition (Figure 5a). The paired oviposited/pinned comparisons performed by a fold change analysis at days 1, 3, and 9 allowed us to tentatively identify the chemical markers (Table S6) and to follow their dynamics along all of the sampling times (Figure 5b). This approach allowed us to explore the chemical dynamics involved in the avocado’s response to oviposition, discarding the mechanical damage caused by the pin treatment. The chemical compounds identified as over- and down-accumulated in the oviposited/pinned samples belong to different chemical groups such as lipids, phenolics, and terpenoids (Table S6), and they are differentially time-regulated. In the early oviposited samples (days 1 and 3) compared to the early pinned samples, there was an accumulation of lipids (monoacylglycerols, obtusilactone A, oleoyl glycine), acetogenins (avocadene acetate, avocadyne and avocadyne acetate), a tocopherol derivative (9’-carboxy-gamma-chromanol)), phenolics (secoisolariciresinol, caffeic acid, scopoletin, acetosyringone and coumaroylquinic acid), terpenoids (gibberellin), and the chlorophyll catabolite pheophorbide A (Figure 5b). In contrast, the early pinned samples exhibited higher contents of glycerophospholipids, the steroid ester campesteryl-18:1, the acetogenins persenone A and persenone B, the phenolics catechin, chlorogenic acid, quercetin and procyanidin C1, and the terpenoids desglucocheirotoxol such as ent-16β-methoxy-19-kauranoic acid. Interestingly, in the late (day 9) oviposited/pinned samples, there is a radical chemical change that is also observed in the PCA (Figure 5a). In the oviposited samples, there is an accumulation of the acetogenins persenone A, persenone B and avocadene acetate, the phenolics proanthocyanidin A, chlorogenic acid and catechin and the diterpenoid desglucocheirotoxol-like compound, and ent-16β-methoxy-19-kauranoic acid. In contrast, in the late pinned samples, there is an accumulation of lipids such as monoacylglycerols, avocadyne acetate obtusilactone A, terpenoids such as gibberellin, and phenolics such as caffeic acid, cinnamtannin A2, and secoisolariciresinol. Furthermore, the integration through linear modeling of the metabolites with transcripts only responding to the oviposition damage resulted in 10 compounds with a positive correlation (Figure 5c, Table S7). The Hass avocado fruit’s responses to the pin, ovi, or both of the treatments are highly diverse and dynamic regarding the secondary metabolism. Nevertheless, the transcriptomics and metabolomics joint analyses allowed us to identify the phenolic pathway as one of the main routes involved in the response to oviposited/pinned Hass avocados. Figure 6 represents its reconstruction based on the homologs/orthologs identified in other plant species and the tentatively identified metabolites.

## 3. Discussion

In nature, *A. ludens* cannot infest *P. americana* (cv. Hass), and Aluja et al. [70] originally reported a mechanism of defense against oviposition, through which the fruit produces neoplasia around the egg mass, thereby killing the eggs (see also p. 14 [72] for additional pictures of the phenomenon). To elucidate the molecular processes involved in this defense mechanism, here, we worked directly in an avocado orchard in the field, observing the response of the Hass avocado fruit to a sterile pin or an *A. ludens* female aculeus in a time course experiment. We are aware that in field experiments, not all of the variables can be controlled, possibly generating a certain degree of noise compared to the experiments that are run under controlled conditions in the laboratory. However, our aim was to mimic and describe/quantify the process of the Hass avocado defense as it happens in nature. We note that when *A*. *ludens* females lay their eggs, some bacteria were likely deposited on the surface of the egg (Figure S1). The vertical transmission of bacteria from the flies to their eggs was documented by Lauzon et al. [79] and more recently confirmed by Majumder et al. [80], among other authors. The response process (to the eggs or possibly the bacteria on them, as well as the physical damage caused by the aculeus insertion into the fruit) can be summarized in four steps, as follows: (1) the release of metabolites in response to the mechanical cell disruption caused by the biotic (aculeus of *A*. *ludens*) or abiotic (sterile pin) element; (2) the oviposition (insertion of egg into the fruit pulp) produces a downstream specific response that is likely mediated by an endochitinase receptor that triggers an immune-like response via the MAP kinase pathway; (3) additionally, other constitutive receptors sense the damage in the cell, triggering a defense response via an oxidative burst producing ovicidal and antibacterial metabolites; (4) finally, cell expansion is triggered in the damaged tissue, generating a neoplasia that encapsulates the eggs.

Based on the number of DEGs identified, it has become clear that the oviposited fruit exhibited a more complex and diverse response than pin-treated ones did; however, it is also true that some of the biological processes mediated by the molecular responses identified could be partially shared in both of the treatments (pin and ovi). This is perhaps because in our experiments, as in others that involve the study of plant defense responses, after the avocado fruits perceived the stimulus of the molecular patterns associated either with damage (DAMPs), the eggs (EAMPs), and/or microorganisms or pathogens (MAMPs or PAMPs) via the specific receptors, these extracellular stimuli were apparently transduced into the cellular responses by plant mitogen-activated protein kinase (MAPK) cascades. The MAPK cascades play a critical role in gene expression, metabolism, cell death, proliferation, and differentiation, and they are evolutionarily conserved among the eukaryotes. In plants, the MAPK cascades are also involved in various biotic and abiotic stress responses, hormone responses, cell division, and developmental processes [81,82]. The wound hormone JA represents a central player in the induced resistance of plants when they are attacked by herbivores or necrotrophic pathogens. It has been argued that this hormone is also involved in the “damaged self-recognition” mechanism, which can be triggered in plants when the surrounding cells in the damaged area perceive the molecular signals of damage, that is, the degraded plant molecules or molecules localized outside their original compartment, and these are perceived as DAMPs [83,84]. The wound-induced responses are both fast, such as the oxidative burst and the expression of defense-related genes, and slow/delayed, such as the accumulation of proteinase inhibitors of hydrolytic enzymes or the synthesis of secondary metabolites. Moreover, it has been reported that the responses to wounding take place both at the site of damage (local response) and systemically (systemic response), and they are mediated by additional hormones such as JA, ET, SA, and ABA [30,85]. It is well known that regeneration in plants largely relies on the coordination of targeted cell expansion and oriented cell division, and these are two biological processes in which the major growth hormone, auxin indole-3-acetic acid (IAA), plays a key role [86,87]. This is consistent with previous reports in which, using *Arabidopsis* roots as a study model, it was proven that auxin is specifically activated in wound-adjacent cells, thereby regulating cell expansion, cell division rates, and regeneration. These wound responses depend on cell collapse of the eliminated cells presumably perceived by the cell damage-induced changes in the cellular pressure [88,89]. Together, this prior knowledge can explain, at least in part, the presence of several genes that are identified as differentially expressed and involved in the synthesis, signaling, and/or response of phytohormones such as JA, ET, SA, ABA, and the auxin IAA, all of which we identified in pin or oviposition damaged fruit, or both of the treatments tested in this study (Table S5).

Our results suggest that there is a specific response of the fruit pulp to an external biological component. Based on our microscopy observations, the response could be possibly triggered by fly eggs or the bacteria surrounding the egg (Figure S1). We found two upregulated genes (UN084237 and UN076164), annotated by Gene Ontology as GO:0009617 and GO:0042742, related to the defense response to the bacteria, suggesting that the latter one could trigger the observed response (Table S4). However, we should be careful with this idea, since we cannot definitively discard the possibility of the occurrence of possible contamination by handling (despite the fact of the extreme care/asepsis under which we worked), and also because we do not know if the number of bacteria on the surface of the eggs was enough to induce the hypersensitive-like response. In this respect, Paniagua-Voirol et al. [90] concluded that the plant’ responses to egg deposition are not induced by egg-associated bacteria, but rather by a secretion attached to the eggs. In addition, we found one gene (UN039405) related to GO:0098542, which is referred to as a defense response to other organisms, two genes (UN086873 and UN088634) related to GO:0009615, which are associated with the responses to viruses, and two others (UN051514 and UN081607) related to GO:0080027, which were labelled as herbivore responses, and four genes (UN047162, UN007801, UN012748, and UN013028) related to the defense response to fungi (GO:0050832). The high levels of transcripts from the Unigene UN047162 strongly called our attention because the partial sequence of this Unigene, annotated as a transcript codifying for an endochitinase B (ChiB), resulted to be 95% identical to Psr a 1, a 32-kDa endochitinase, which is considered to be one of the major allergens of avocado [91] and which belongs to Group three of the pathogenesis-related proteins (PR-3) in the classification of Stintzi et al. [92]. These endochitinases are part of the plant’s basic defense system against fungal pathogen attacks. Interestingly, in *Arabidopsis*, it has been shown that CERK1, a membrane receptor belonging to the LysM receptor family involved in chitin/chitosan binding and knock-out mutants on these receptors, shows neither a reactive oxygen burst nor MAP kinase activation in the response to chitosan [93]. Despite there being no homolog to the LYS receptors and nucleotide-binding leucine-rich repeat (NLR) family receptors having been identified as differentially expressed Unigenes, many of them (27 LYS receptors and 486 NLR receptors) were identified in the transcripts dataset generated in this study (Tables S8 and S9). However, we cannot conclusively determine if the egg, the adult insect via its aculeus, or bacteria on the egg’s surface triggered the response.

Cellular responses to stimuli such as wounds quickly generate an oxidative burst, which is activated through the calcium-protein kinase C signaling pathway, leading to increased ROS production, which in this case, is mediated by the activity of NOX2, an NADPH oxidase enzyme. This is consistent with the identification of two DEGs, the Unigenes UN086873 and UN088634, both of which are homologues to AT3G51440, a calcium-dependent phosphotriesterase superfamily protein, which is involved in the responses to ET, fungi, JA, SA, and wounding [94], and with the presence of several Unigenes coding to FAD/NAD(P)-binding oxidoreductase family proteins (UN022519, and UN030168). Despite acting intra-cellularly, ROS, in conjunction with antioxidant enzymes, play a key role in turning enzymes on and off, acting like a second messenger. High levels of ROS can lead to cellular damage, oxidative stress, and DNA damage, which can elicit either cell survival or apoptosis mechanisms depending on the severity and duration of the exposure. We also noticed that mitogenic signaling begins at the cell surface with the ligand-dependent activation of receptor tyrosine kinases, which activate important MAP kinase cascades necessary for proliferation. These cascades lead to the generation of H_2_O_2_ from several enzyme catalysts, including the NADPH oxidases. Several other genes, not only the Unigenes UN022519 and UN030168 (which codify to FAD/NAD(P)-binding oxidoreductase proteins), which are involved in the responses to oxidative stress or cellular oxidant detoxification were identified as DEGs (Table S5) either in both of the treatments or only in the oviposition treatment, such as glutathione peroxidase (gpx, ID: UN109125), which is part of the arachidonic acid, xenobiotics biodegradation, and glutathione metabolism. In addition, a plant cysteine oxidase (PCO, ID: UN041269) was up-regulated. It has been reported that gpx and PCO are related to an improved capacity to respond to environmental and biotic stress, reacting to auxins and ET [95,96], sensing and regulating the redox condition, and participating in the rebalance process after the oxidative burst. Here, we suggest that in sterile pin treatments, the oxidative burst caused by the wound or damage was quickly counteracted by several mechanisms, which include both local and systemic responses targeted at regulating cell expansion, cell division rates, and regeneration. For its part, the damage caused by the aculeus of female flies in ovi treatments provides nutrients to the microorganisms/pathogens present in the oviposited eggs and facilitates their entry into the tissue and maybe its subsequent infection.

The presence of some of these metabolites differentially accumulated in the early (days 1 and 3) and late periods (days 6 and 9) in both the ovi and pin samples is consistent with biological activities which have been reported for many of these compounds, and the role that we suggest they may play in the immunity response to oviposition is mainly triggered by the molecular patterns such as EAMPs, and MAMPs or PAMPs. For example, acetogenins such as avocadene acetate, avocadyne, and avocadyne acetate (synonyms for 1-acetoxy-2,4-dihydroxy-n-heptadeca-16-ene, 1,2,4-trihydroxyheptadec-16-yne, and 1-acetoxy-16-heptadecyne-2,4-diol, respectively), which were mostly accumulated in the ovi samples early in the experiment, are antifungal compounds whose activity involves the quiescence of the germinated appressoria of *Colletotrichum gloeosporioides* [97,98,99,100]. Phenolic compounds such as secoisolariciresinol, caffeic acid, and some *p*-coumaric acid derivatives exhibit antioxidant and/or antimicrobial activities [101]. 

In addition, proanthocyanidins, the major bioactive chemical constituents in phytoalexins present in *Camellia sinensis* leaf extracts, are responsible for the larval mortality of the most prolific Afrotropical malaria vectors (*Anopheles arabiensis* and *A. gambiae*) [102]. We note that not only the compounds such as secoisolariciresinol and caffeic acid [101], but also chlorogenic acid [103], are potent antioxidants, suggesting that the immune response as well as the damage response (DAMs) involve ROS production, which may act as a signal transducer, but also, at some point, biosynthesized antioxidant compounds that can reduce the oxidative stress to improve the immune function [104].

Furthermore, our metabolomics study identified that *p*-coumaric acid (p-CA) was negatively correlated with the transcripts exclusively associated with oviposition. p-CA is known to be part of the plants’ defense repertoire against pathogenic bacteria and fungi [105,106,107], but it has also been reported as an antagonist of the MAPK pathway, inhibiting the protein kinases and avoiding the neoplastic growth in plants and mammals [108,109]. In our study, two auxin response factors (ARFs) with a B3 DNA-binding domain (DBD) were down-regulated in the oviposition treatment. In tomatoes, the down-regulation of the ARFs produces changes in the structure of the pericarp tissue, promoting cell division and increasing its firmness [110], regulating cell wall expansion [111]. In addition, we tentatively identified pheophorbide A, a key chlorophyll catabolite in the early oviposited samples (Figure 5b, Table S6). Interestingly, the accumulation of phephorbide A induces light-independent cell death in the leaves of *A. thaliana*, and it has been suggested that this compound may function as a signal molecule regulating gene expression and inducing cell death programs [112]. Our results support the above hypothesis, since the accumulation of pheophorbide A was observed only in early stages after oviposition (Figure 5b and Table S6).

Our results also suggest that the pattern-triggered immunity (DAMPs, EAMPs, MAMPs, and/or PAMPs), including the effector-triggered immunity (effectors not identified yet) observed, could explain the differences in terms of the number and classes of transcription factors specifically identified in the pin or ovi treatments (Table S5) and the accumulation of some of the secondary metabolites that possess insecticidal activity. Despite the fact that little is known about the dynamics of the cellular and subcellular localization of defensive phytochemicals during encounters with herbivorous insects or microbial pathogens, and precise knowledge of their mode of action is still scant, it has been suggested that at least some of these insecticidal or antibiotic compounds may be involved in controlling several immune responses that are evolutionarily conserved in the plant kingdom, including callose deposition and programmed cell death [113].

**Figure 7:**
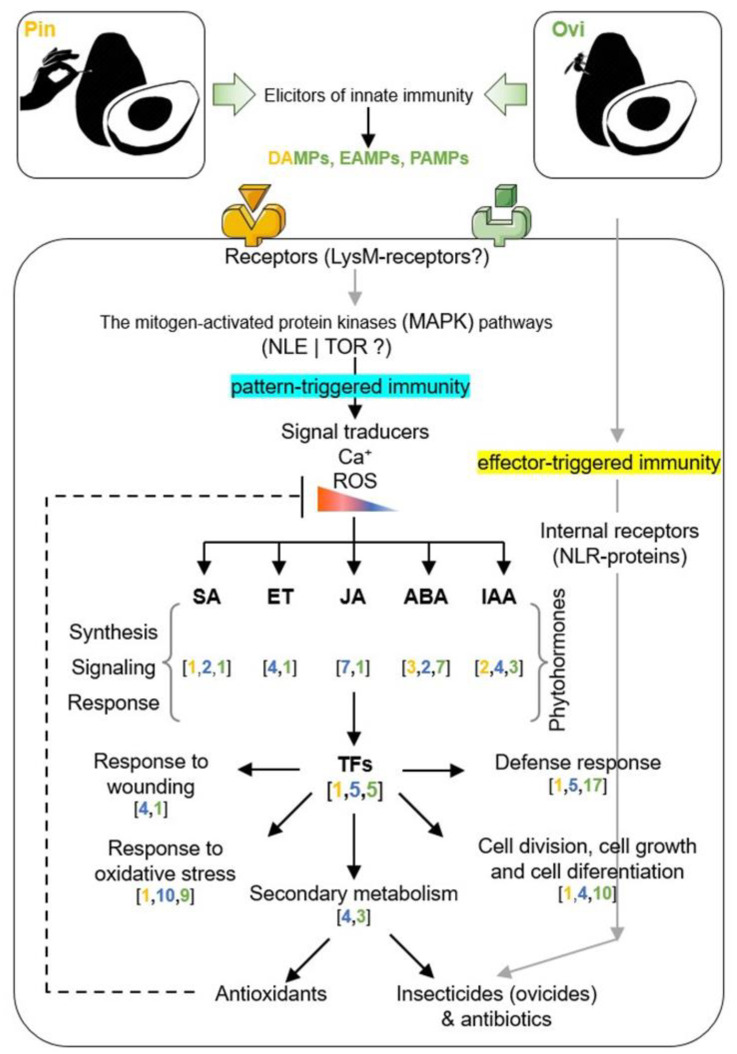
Diagram for the hypothesized molecular response mechanism in *P. americana* (cv. Hass) triggered after wounding using a sterile pin (pin) or oviposition by *A. ludens* females (ovi). Differentially expressed Unigenes (DEG) identified in pin, ovi, or both treatments are shown in brackets, and they are highlighted by yellow, green, or blue colors, respectively. Biological processes in which DEGs are involved are also represented. Black arrows indicate connections and signaling pathways of the functional categories or biological processes involved in pin or ovi responses and which are backed by DEGs. Gray arrows indicate putative categories or processes. Dotted line indicates the antioxidant properties that may regulate biosynthesized compounds to reduce oxidative stress as a mechanism to improve immune function. MAPK = Mitogen-activated protein kinase, NLE = Notchless Homolog, TOR = Target of Rapamycin, ROS = Reactive Oxygen Species, NLR = Nucleotide-binding and leucine-rich repeat immune receptors, SA = Salicylic acid, ET = Ethylene, JA = Jasmonic acid, ABA = Abscisic acid, IAA = Indole Acetic Acid, TFs = Transcriptional Factors.

The molecular mechanism related to the ability to form a neoplasia and encapsulate single eggs or egg masses (clutches) in an oviposition-induced plant response is almost unknown, except for what has been reported in the pea *P. sativum*. The pea plant senses the oviposition fluid of *B. pisorum*, which contains bruchin that induces neoplastic growth, precluding the development of the larvae [41,114]. Recently, three genomic sites associated with HR-type cell death induced by eggs were reported in *Brassica rapa*; these regions contain cell surface receptors, intracellular receptors, and genes related to the immune response [115]. In our study, we observed cell expansion (Figure 1), which resulted in neoplasia formation such as that which was reported by Aluja et al. [70] and the one caused by the fruit spotting sting bug sting [72]. We identified transcripts related to oviposition by *A*. *ludens* females, observing a direct and immediate molecular response against the eggs presence 20 min after the experiment, triggering a chitin receptor (ChiB). This receptor could sense chitin, which is a component of the fungal cell walls and is also present in the exoskeleton of insects [116] and various insect structures such as the aculeus [117], which is considered to be a PAMP [118,119]. It has been suggested that chitin is not present in eggshells [44]; however, the metabolic pathways involved in the production of chitin start in the early stages of *D. melanogaster* development, while the presence of a chitin-like component was identified in the eggshells and eggs of *Aedes aegypti* (Diptera: Culicidae) [120,121,122,123]. Based on the latter one, it is possible that Hass avocados could detect the presence of chitin in the aculeus and eggshells/chorion of *A. ludens*. ChiB was homologous to K20547 in the KEGG database, which belongs to the plants’ mitogen-activated protein kinase pathway (MAPK). So, ChiB seems to activate the defense mechanism through the MAPK pathway. Moreover, ChiB had high homology with the endochitinase class II of *Carica papaya*. This enzyme has been found abundantly in the laticifers of *C. papaya* [124], a latex component produced by papaya that, besides other proteins, has insecticide and antifungal properties, with the ability to break down chitin [125], and it is associated with the immune response activation [126]. This enzyme has been observed to be induced as a response to fungi in cucumber (*Cucumis sativus*) and *P*. *americana* [91,127]. In the latter one, the inhibition of the growth and branching of the fungus was observed, and this supports our hypothesis that a response mediated by the sense of chitin elicits the production of secondary metabolites with antimicrobial properties (Figure 7).

## 4. Materials and Methods

### 4.1. Plant Material and Treatments

As our goal was to study the molecular defense mechanism triggered by *P. americana* cv. Hass when *A. ludens* females inserted an egg mass into the fruit, and two conditions were contrasted: a fruit, into which a female fly inserted its aculeus into the mesocarp through the exocarp and deposited eggs (hereafter known as ovi), and a fruit damaged with an aculeus proxy (a sterilized entomological pin (hereafter known as pin)), without the deposition of eggs. The mean size (length) of the five aculei we measured was 3.64 ± 0.127 mm, and the width was 0.112 ± 0.004 mm. In the case of the pin (also five measurements), the values were 4.49 ± 0.24 mm (length) and 0.169 ± 0.007 mm (width), respectively. First, we surveyed the structural changes at the histological level which occurred in the Hass avocados at 1, 20, and 40 days after the treatment. This was based on the original study by Aluja et al. [70], and we documented the formation of a neoplasia (hardened callous tissue) surrounding the egg masses deposited by the *A*. *ludens* females into the Hass avocados. Subsequently, we performed an additional study to identify the molecular mechanisms at play shortly after the damage was inflicted using the sterile pin or the aculeus of the *A*. *ludens* females (day one) and also three, six, and nine days after the damage (details of the transcriptomics and metabolomics analyses can be found in Section 4.3 and Section 4.6). For each time point, three replicates were performed, with each replicate stemming from a different tree. The experiments were performed in an avocado orchard located in Champilico, Altotonga, Veracruz, Mexico (19°45′29.23″ N and 97°15′19.06″ W), which was located at 1926 m.a.s.l. The avocados had reached commercial maturity, with 275.8 ± 55.7 (mean ± standard deviation) g fresh weight, and they were 10.99 ± 1.1 cm in length, 7.22 ± 0.5 cm in diameter, the firmness was 358.8 ± 22.6 N (fruit with peel), and the dry weight was 32.54 ± 3.9%. The experimental fruit were covered with cloth to protect them from any type of damage (insects/bird/pathogen attacks) several weeks before the experiment started.

Prior to the beginning of the experiment, we thoroughly rinsed the Hass avocados attached to the tree branches/twigs with sterile distilled water and placed an observation device on them. This device consisted of a transparent cone-shaped plastic container with a proximal diameter of 4 cm, a distal diameter of 2.2 cm, and a height of 3 cm. Inside the device, we placed two 15–20-day-old, mated/gravid *A. ludens* females, and we observed them until they had laid a clutch of eggs (this was confirmed by aculeus dragging/host marking; [128]). From the previous study by Aluja et al. [70], we knew that wild *A*. *ludens* females laid 11.2 ± 0.7 eggs per clutch when forcibly infesting Hass avocados. To cause the pin damage, we inserted the 0.169 mm (width) and 4.49 mm long sterilized pin into another fruit in a separate branch. The exact locations of the fly aculeus insertion (oviposition) and the pin damage were marked using a blue Sharpie pen (Newell, Atlanta, GA, USA) dot. To retrieve the tissue samples, we used a disinfected 0.5 cm diameter stainless steel punch, which we inserted into the marked location. The samples were retrieved minutes after the oviposition/pin damage (sampling time 1), and after 20 and 40 days, they were immediately transported to INECOL’s laboratories in a cooler with ice to avoid oxidation.

### 4.2. Microscopy

To visually determine the differences between the Hass avocado responses to the biological (aculeus of female *A*. *ludens* and the eggs deposited into the fruit pulp via this “tube”) and non-biological objects (sterilized pin) over a time course period of 1, 20, and 40 days, we used various microscopy techniques, as follows.

#### 4.2.1. Optical Microscopy

The samples were fixed depending on the type of microscopy technique used (details follow), then, cross sections were made at the site of oviposition/pin damage. Images were taken using a stereomicroscope Carl Zeiss SteREO Discovery.V8 coupled to color 105 Axiocam camera (Carl Zeiss Meditec AG, Jena, Germany) using an Achromat 0.5 × FWD 134 mm objective (Carl Zeiss).

#### 4.2.2. Scanning Electron Microscopy (SEM)

The samples were fixed in a Karnovsky solution for 48 h, washed three times with Sorenson’s buffer (pH 7.2), and dehydrated gradually in 30, 50, 70, and 96% ethanol (Cat. 5405-20L, MEYER, MEX) for two h in each concentration and 100% ethanol (Cat. 9000-02, J.T. Baker, USA) for 30 min, three times. Then, the samples were dried at a critical point using a Quorum K850 dryer (Quorum Technologies Ltd., Asfford, England) that was mounted on conductive carbon tape and was finally coated with gold according to Bozzola and Russel [129]. The observation and acquisition of the micrographs was achieved using a scanning electron microscope FEI Quanta 250-FEG (FEI Inc., Valley City, ND, USA).

#### 4.2.3. Confocal Microscopy

The samples were fixed with 4% *p*-formaldehyde that was prepared in a sodium phosphate-buffered solution (PBS; pH 7.2), and then washed with distilled water. For the staining, we used acridine orange for 5 min, and calcofluor-white for 10 min. A Leica TCS-SP8+STED microscope (Leica Microsystems CMS GmbH, Mannheim, Germany) was used with the following configuration: TileScan merging (10X/NA = 0.3, zoom 0.75) and xyz (63X/NA = 1.40 oil, zoom). The calcoflour (434–479 nm, gray channel), acridine orange (541.577 nm, red channel), and reflection (479–498 nm, yellow channel) channels were activated for the recordings.

### 4.3. Transcriptomics Analysis

Based on the microscopy results, we reduced the sampling interval to capture the early transcriptomics alterations that occurred in the Hass avocados after the ovi/pin treatments. For this, we repeated the ovi/pin treatments using the same conditions. However, in this case, the samples were collected 1 (20 min after treatment), 3, 6, and 9 days after the oviposition (ovi_1, ovi_3, ovi_6, and ovi_9) or pin (pin_1, pin_3, pin_6, and pin_9) treatments. Non-damaged fruits were invariably included as controls. For each condition, three replicates were considered, with each fruit stemming from a different tree. The samples were obtained as previously described, transported in liquid nitrogen from the field to the laboratory, and then stored at −80 °C until processing (same procedure used to obtain the samples for the metabolomics analysis described later).

The samples pulverized with liquid nitrogen were used for the RNA extraction following a scaled-down protocol that has been described previously [130]. The RNA integrity was evaluated by capillary electrophoresis using a Bioanalyzer 2100^®^ (Agilent Technologies^©^, Santa Clara, CA, USA). The RNA concentration was measured by fluorometry using a Qubit 2.0^®^ (ThermoFisher Scientific^©^, Waltham, MA, USA). Sequencing libraries were generated using the TruSeq RNA library preparation kit^®^ (Illumina Inc.^©^, San Diego, CA, USA). The library validation was performed in a Bioanalyzer 2100^®^ (Agilent Technologies^©^, Santa Clara, CA, USA) and used for sequencing in a NextSeq500^®^ platform (Illumina Inc.^©^, San Diego, CA, USA) in a 150 bp paired-end format.

### 4.4. Read Processing, Assembly, and Functional Annotation

The raw reads were filtered using a Python script “https://github.com/Czh3/NGSTools/blob/master/qualityControl.py (accessed on 1 August 2022)” to keep the reads with at least 25 on the Phred quality score in 80 percent of the sequence. The resulting paired filtered reads were trimmed and overlapped into single longer reads using SeqPrep “https://github.com/jstjohn/SeqPrep (accessed on accessed on 1 August 2022)”. The filtered reads were assembled using the Trinity v2.0.2 pipeline [131]. Then, the longest isoform of each contig was recovered and considered as the Unigenes. The PolyA sequences were removed by using SeqClean “https://sourceforge.net/projects/seqclean/files/ (accessed on accessed on 1 August 2022)”. The sequences from other organisms were removed using DeconSeq “https://sourceforge.net/projects/deconseq/files/ (accessed on 1 August 2022)” by comparing the unigene list against the databases of bacteria, virus, insects, and fruit flies. The open reading frames (ORFs) were predicted using AlingWise and by employing a custom database conformed by 11 plant proteomes downloaded from RefSeq database, including *Amborella trichopoda* (NCBI:txid13333); Monocots: *Musa acuminata* (NCBI:txid4641), *Oryza sativa* (NCBI:txid4530), *Zea mays* (NCBI:txid4577) and *Sorghum bicolor* (NCBI:txid4558); Eudicots: *Vitis vinifera* (NCBI:txid29760), *Solanum lycopersicum* (NCBI:txid4081), *Prunus persica* (NCBI:txid3760), *Populus trichocarpa* (NCBI:txid3694), *Arabidopsis thaliana* (NCBI:txid3702) and *Theobroma cacao* (NCBI:txid3641). Finally, BlastClust “https://ftp.ncbi.nlm.nih.gov/blast/documents/blastclust.html (accessed on 1 August 2022)” was used to reduce the redundancy of the Unigenes.

### 4.5. Identification of Differentially Expressed Genes (DEGs)

The RSEM pipeline [105] was used to obtain the read counts in terms of transcripts per million (TPM). The assembled, filtered transcriptome was used as a reference for the gene expression analysis. The quality filtered reads of each sample were mapped onto the reference using bowtie2 software [132], and the number of read counts that were mapped onto each gene was normalized by comparing the pairs of samples using DESeq2 [133]. As we assayed in the field directly in an avocado orchard in a time course fashion, we expected some not controlled source of variation; therefore, we performed an analysis of the variance partition with the differential expression for the repeated measures (dream) from the Variance Partition package [134], which uses linear models (lm) to determine the gene-wise variance related to the treatments. Briefly, the tximport package [135] was used to load the data in the R environment, and the counts were normalized with the DESeq2 [133] by the library size correction scaling factors. A gene expression cutoff was set up, and the genes with less than a sum of one fragment per million in less than 50% of the samples were filtered out. The formula for the lm was set up as “~ Treatment + Days”. The genes with at least 30% of variance explained by the treatment and an FDR *p* value < 0.05 in the linear model were considered to be differentially expressed.

The annotation of the DEGs was performed using the BlastKOALA (with the plants genomes) [136], eggNOG-mapper [137], and with unidirectional BLASTP best hit analyses using the *Arabidopsis* proteome as a reference. The Gene Ontology (GO) terms were inherited to *P. americana* genes mainly based on their identified *A. thaliana* homologs. The DEGs annotated with KOs were mapped onto the KEGG metabolic pathways using the KEGG Mapper-Reconstruct application [138].

### 4.6. Metabolomics Analysis

The methanolic extracts for each sample, 1 (20 min after oviposition), 3, 6, and 9 days after the oviposition (ovi_1, ovi_3, ovi_6, and ovi_9) or pin (pin_1, pin_3, pin_6, and pin_9) treatments were obtained by using an accelerated solvent extraction system (ASE 350, Dionex, Thermo Scientific^©^, Waltham, MA, USA) as previously reported [139,140]. The untargeted metabolomics analyses were performed using four replicates per treatment in an ultra-high resolution liquid chromatograph coupled to a high-resolution mass spectrometer (Class I, Synapt G2-Si, HDMI, Waters™, Milford, MA, USA) according to Monribot-Villanueva et al. [139,140]. The data were processed using Waters™ MassLynx software (Version 4.1), and the statistical analyses were run using MetaboAnalyst software “https://www.metaboanalyst.ca (accessed on 2 June 2022)” as described in Chong et al. [141]. Tentative identification was performed by using Metlin “https://metlin.scripps.edu (accessed on 2 June 2022)” and FooDB “http://foodb.ca/ (accessed on 2 June 2022)”, and a value of ± 5 ppm was the maximum mass error that was allowed.

## 5. Conclusions

In conclusion, we have shown that the response of Hass avocados to the oviposition of *A. ludens* triggers an immune-like response that kills the eggs by producing secondary metabolites with potential ovicide and antimicrobial effects, such as avocadyne and avocadene. This indicates that the plant quickly senses the presence of the eggs via a chitin receptor and/or possibly the bacteria attached to the eggs activate the MAPK pathway. The plant also responded by generating a neoplasia, encapsulating the eggs, a process that likely also contributed to their demise through desiccation (see also Aluja et al. [70], Figure 1). However, some questions remain regarding which molecule elicits the response and how specialized this mechanism is regarding other fruiting trees. Finally, the presence of the potential ovicide metabolites opens the possibility of inducing these compounds in the plant to fend off the attacks of herbivores. Furthermore, given that the defense mechanism detected is apparently a generally conserved mechanism that some plants developed millions of years ago [75], our findings could have broader implications for pest andalso possibly pathogen management.

## Data Availability

The raw datasets for this study can be found in the Sequence Read Archive (SRA) of the National Center for Biotechnology Information (NCBI) under the accession number PRJNA551035 “https://www.ncbi.nlm.nih.gov/sra/?term=PRJNA551035 (accessed on 17 January 2023)”.

## Supplementary Materials

The following supporting information can be downloaded at: https://www.mdpi.com/article/10.3390/ijms24032060/s1.
