# Supplementary material for: Assessment of the Molecular Responses of an Ancient Angiosperm against Atypical Insect Oviposition: The Case of Hass Avocados and the Tephritid Fly Anastrepha ludens
Source: Int J Mol Sci. 2023 Jan 20;24(3):2060. doi: 10.3390/ijms24032060 (PMC9916504; doi:10.3390/ijms24032060)
Supplement: Supplementary file 1 [file ijms-24-02060-s001.zip › Supplementary Figure S1.pdf]

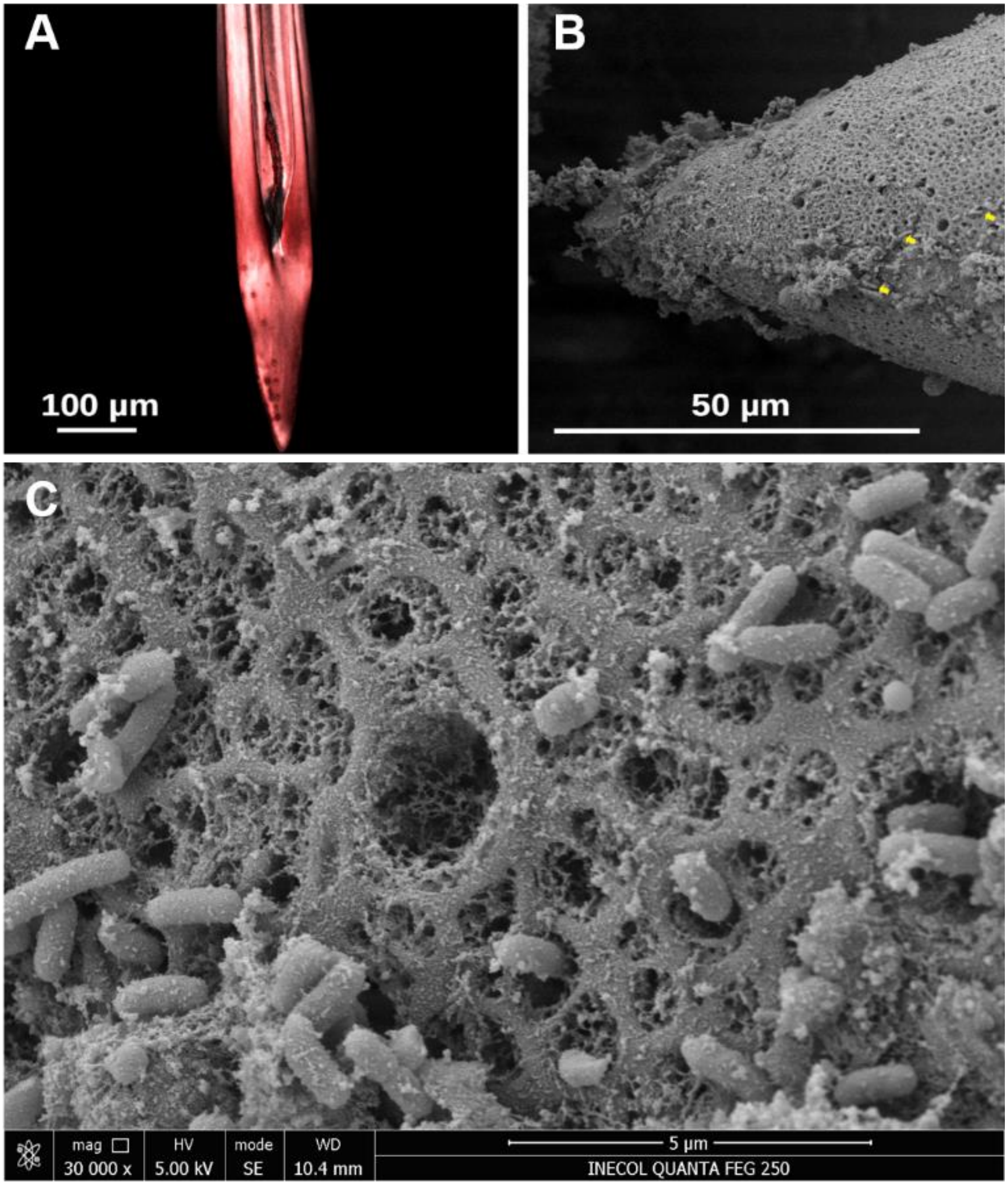

Supplementary Figure S1. (**A**) Confocal microscopy image showing an aculeus of an *Anastrepha ludens* female. (**B**) SEM images of the surface of an *A. ludens* egg depicting bacteria-like structures (yellow arrows). (**C**) Magnification of multiple bacteria-like structures on the egg surface.
